# Automated Discovery of Novel Drug Formulations Using Predictive Iterated High Throughput Experimentation

**DOI:** 10.1371/journal.pone.0008546

**Published:** 2010-01-01

**Authors:** Filippo Caschera, Gianluca Gazzola, Mark A. Bedau, Carolina Bosch Moreno, Andrew Buchanan, James Cawse, Norman Packard, Martin M. Hanczyc

**Affiliations:** 1 ProtoLife Inc., San Francisco, California, United States of America; 2 Institute of Physics and Chemistry, University of Southern Denmark, Odense, Denmark; 3 Santa Fe Institute, Santa Fe, New Mexico, United States of America; 4 European Center for Living Technology, Venice, Italy; 5 Reed College, Portland, Oregon, United States of America; 6 Initiative for Science, Society, and Policy, University of Southern Denmark, Odense, Denmark; 7 Cawse and Effect, Pittsfield, Massachusetts, United States of America; University of Vermont, United States of America

## Abstract

**Background:**

We consider the problem of optimizing a liposomal drug formulation: a complex chemical system with many components (e.g., elements of a lipid library) that interact nonlinearly and synergistically in ways that cannot be predicted from first principles.

**Methodology/Principal Findings:**

The optimization criterion in our experiments was the percent encapsulation of a target drug, Amphotericin B, detected experimentally via spectrophotometric assay. Optimization of such a complex system requires strategies that efficiently discover solutions in extremely large volumes of potential experimental space. We have designed and implemented a new strategy of evolutionary design of experiments (Evo-DoE), that efficiently explores high-dimensional spaces by coupling the power of computer and statistical modeling with experimentally measured responses in an iterative loop.

**Conclusions:**

We demonstrate how iterative looping of modeling and experimentation can quickly produce new discoveries with significantly better experimental response, and how such looping can discover the chemical landscape underlying complex chemical systems.

## Introduction

Formulation of a stable lipid membrane with good drug complexation characteristics is a complex experimental problem as there are many possible components (qualitative variables) with a wide range of relative amounts (quantitative variables) in the recipe. This makes drug formulation a good test case for methodologies for discovering and optimizing complex chemical systems. The experiments we present have five qualitative factors and nine components whose concentrations are varied in the aqueous phase, to form an experimental space that contains a total of 82,950 possible experiments.

The experimental approach to the optimization problem considered here is iterated high-throughput experimentation. Many experiments are performed in parallel (e.g., using a 96-well plate format), the results are analyzed, and a successive generation of experiments is performed, with the goal of progressively better results with each generation. After each generation of experiments, the experimenter is faced with the problem of designing a limited yet informative set of experiments from an expansive experimental space for the successive generation.

**Figure 1:**
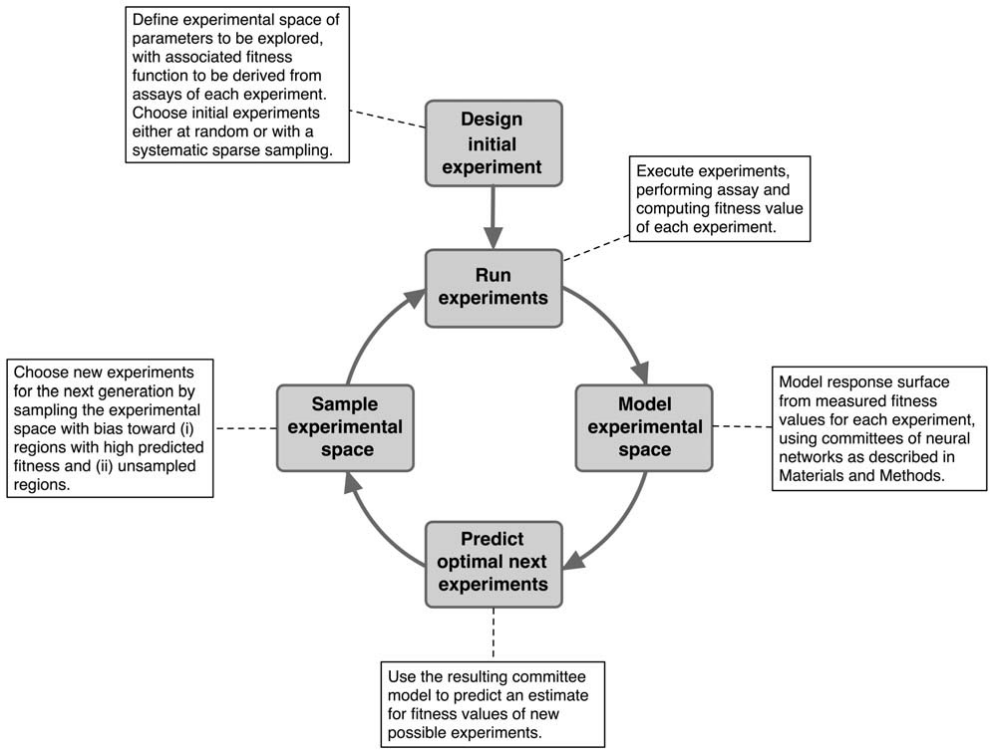
Illustration of the iterated high throughput experimental process. Note the predictive modeling procedure in the loop.

We address the problem of designing experiments for the exploration of high-dimensional experimental spaces by applying statistical modeling and predictive methods to the entire experimental space at each generation. We build models from the raw experimental data, and we use those models to choose where next to sample the experimental space. This methodology is iterated as illustrated in Figure 1. Our procedure is a form of evolutionary design of experiments (Evo-DoE), building on previous work based on genetic algorithms, where experiments in each generation were specified by a genetic code, using genetic operators (mutations and crossovers) to generate new experiments for each successive generation [1]–[5]. The approach used here differs primarily in that new experiments are chosen not only by random variation, but also based on statistical modeling.

To start the optimization process, we sparsely sample the experimental space with a random selection of experiments. We build models of the desired response from the experimental data, design the next sparse sampling of the experimental space, and the process repeats. Tightly coupling experiments with statistical modeling and predictive algorithms enables successful optimization of desired chemical behavior of supramolecular structures in large high-dimensional experimental spaces.

The problem of designing each successive generation of experiments is difficult for two related reasons: the statistical uncertainty of any predictive model given the relatively small amount of data (∼10^2^–10^3^ samples), and the size of the space (particularly when the number of dimensions (experimental variables) is large). Large regions of the space are inevitably unrepresented in the experimental data. To address the first difficulty, we use a bootstrapping procedure in the model-building process to ensure that the model's complexity is not excessive given the quantity of data available. The second difficulty is handled by combining model-based prediction of experiments with a weighted random sampling of the experimental space, where the distribution for the random sample is biased toward the unsampled regions of the space. Given these two difficulties, our approach to the experimental design problem may be seen as an example of establishing an *exploration/exploitation tradeoff* [6], and is a high-dimensional analogue of the technique known as *Kriging* in geostatistics [7].

Traditional approaches to DoE for high-dimensional experimental spaces use a “screening design” to identify a few significant factors of many potential ones, or to identify a few significant factors that embody the “main effects” that are presumed to be an order of magnitude more important than “interaction effects” [8]. Screening designs are typically highly fractionated two-level (and, very rarely, three-level) factorial designs. This approach fails for complex systems because the interactions are typically not first order, but second order. Systems that have several significant qualitative factors with more than two levels, mixture systems crossed with factorial systems, and other such complex designs cannot be fractionated to implement a screening design. Conventional DoE software does not include facilities to code systems that are several sub-designs crossed with each other, like the one described here, which is seen below to be approximately a 2×7 factorial crossed with a 2^2^ factorial crossed with a 3-component constrained mixture crossed with a constrained multilevel qualitative factorial.

Amphotericin B is an antifungal drug used to treat systemic fungal infections. The drug can cause nephrotoxicity if present in high doses. However, when intercalated within a lipid membrane, the hydrophobic drug can be administered to a patient with minimal toxic effects. There are currently three different lipid formulations of Amphotericin B on the market [9]. Each formulation consists of different lipids associated with the drug molecule, indicating that there is more than one way to effectively package the drug into a lipid structure. Of these three, AmBisome [10], [11], has been the most effective and profitable.

In experiments presented here, we describe an Evo-DoE procedure to search within a defined space of 82,950 possible experiments for lipid combinations that maximize the amount of Amphotericin B entrapped in the formulation. In a space of this size and complexity, exhaustive screening is impractical, conventional DoE has severe difficulties handling the multiple qualitative variables, and simple hill-climbing approaches tend to fail because of the interaction effects in the system. Within a few iterations (exploration of ∼0.5% of the space) we found many new promising Amphotericin B formulations not previously reported. In addition we were able to determine experimentally the response surface for the lipid-drug combinations.

## Results

Evo-DoE is an iterated, cyclical process involving experimentally measuring responses of sparsely sampled recipes from a space of possible experimental recipes. One approach to implementing evolutionary DoE is to use a genetic algorithm, which assigns each possible experiment a genetic code, and then progresses from one generation of experiments to the next by applying genetic operators analogous to mutation and crossover to winning recipes [1]–[5]. Here, we explore the experimental space by modeling the entire response surface on the basis of the collected experimental data, and then use that model to choose the next set of recipes to test experimentally. The models used here are feed-forward, single hidden-layer artificial neural networks [12].

The Evo-DoE process used here started with a set of 90 randomly selected recipes that included all the pairwise combinations of lipids in our lipid library. The N^th^ iteration of the Evo-DoE process consisted of the following steps:

1. Measure the experimental response of the N^th^ set of recipes;
2. Optimize the metaparameters of the N^th^ model, as described below;
3. Build a model of the entire response surface from the experimentally measured responses for the first N sets of recipes;
4. Randomly choose 48 recipes from the untried recipes with the top quartile of responses predicted by the model, and add those recipes to the N+1^th^ set of recipes;
5. Add 12 more randomly selected untried recipes to the N+1^th^ set of recipes.

Neural network metaparameters are often optimized by a “bootstrapping” process [13]. At step 2 of the Evo-DoE cycle, model metaparameters were dynamically optimized via the following procedure: Models with different configurations of metaparameters were trained on 20 different datasets, each one a random sample of 70% of the experimentally observed responses. The models were then tested on the remaining 30% of the responses. The accuracy of a given model (correlation of out-of-sample predictions with measured responses) was computed as the accuracy averaged over the 20 different data sets. Finally, the metaparameter configuration that gave the highest accuracy was selected.

The response of each recipe was calculated as follows: Three spectrophotometric absorbance measurements were taken from experimental replicates in three separate wells of a 96-well plate at three different times, and the response of a recipe was defined as the average of those nine measurements.

**Figure 2:**
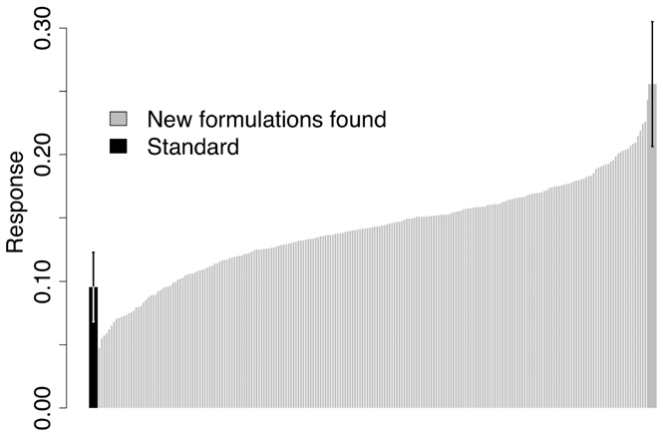
Rank order of all tested formulations found with Evo-DoE vs. the standard recipe. Error bars on the best new recipe were taken from three repeats performed on the same day (space repeats, see Materials and Methods). The error bars from the standard were taken from 75 total repeats performed over the course of the experiment.

The system was quickly optimized (Figure 2), after individually testing 450 individual recipes from a space hundreds of times larger. Even with a fairly substantial amount of noise, common in real chemical systems, a clear optimum was reached as subsequent generations thoroughly explored the same subregion without resulting in further increased response. The amount of noise is apparent from the spread of points for the standard: The control recipe replicated each generation The optimum has a response roughly twice that of the standard, indicating that the recipes have more Amphotericin B in the lipid phase. The standard was prepared using the AmBisome recipe [10], [11], but with our protocol.

**Figure 3:**
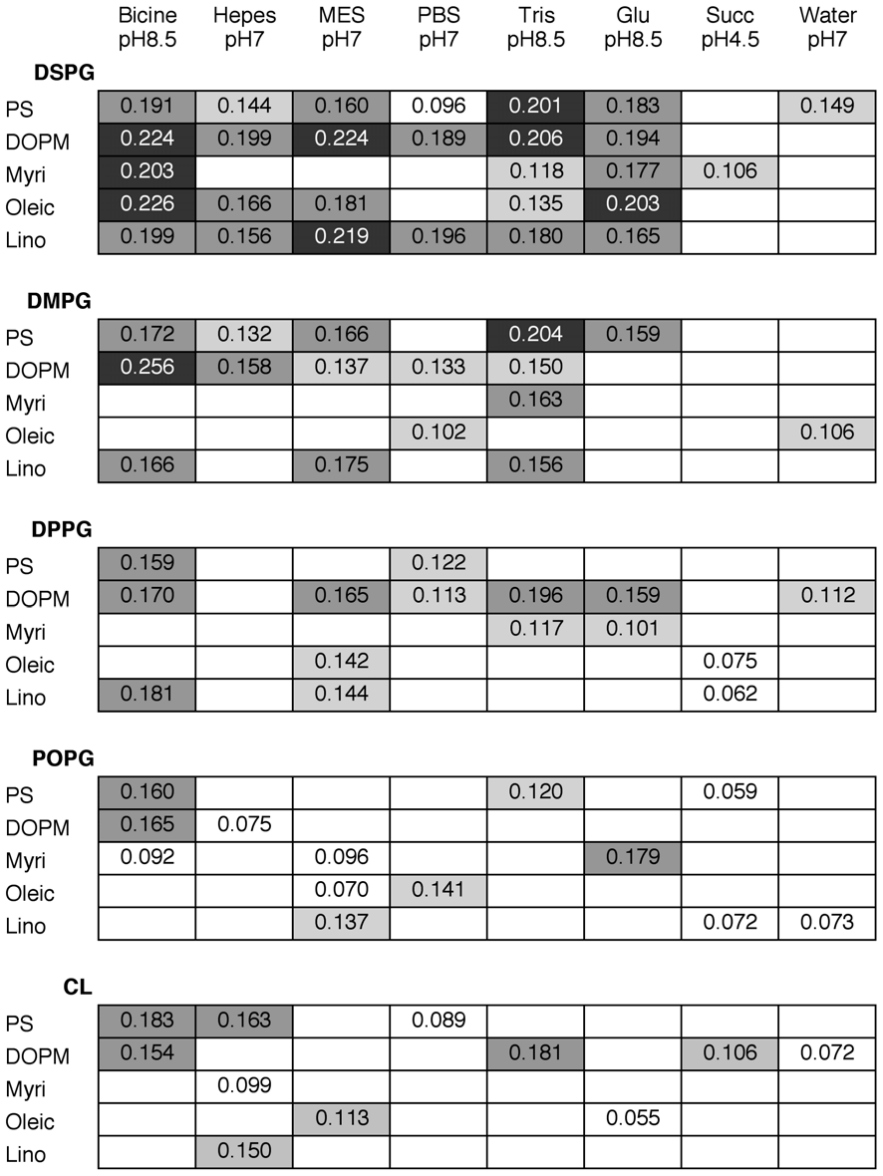
Representation of a 3-dimensional section of experimental space. For each PG-type lipid, shown in bold, the horizontal section lists the lipids from the group with a net negative charge and the vertical section lists the reagents in the aqueous phase and their corresponding pH values. Response levels (the UV/Vis absorbance of Amphotericin B associated with the formulation): dark grey, >0.20; medium grey, 0.15–0.20; light grey, 0.10–0.15; white, <0.10; Blank cells, not determined. For abbreviations, see Materials and Methods.

More detailed analysis of the high response optimum found by Evo-DoE is shown in Figure 3 as a set of 2-dimensional sections of one 3-dimensional section of the entire space.It was generated from a Microsoft Excel pivot table based on the three most important factors found through our Evo-DoE: PG-type lipids, negatively charged lipids, and aqueous factors (complete identification of lipids and aqueous factors is given in the Materials and Methods section). The numbers in each cell are average response values, and the cells are color coded (with three color levels and blank cells). From this representation it is clear that there are substantial high response ridges for components DSPG, Bicine, and DOPM, with lesser ridges for linoleic acid, PS, and oleic acid.

**Figure 4:**
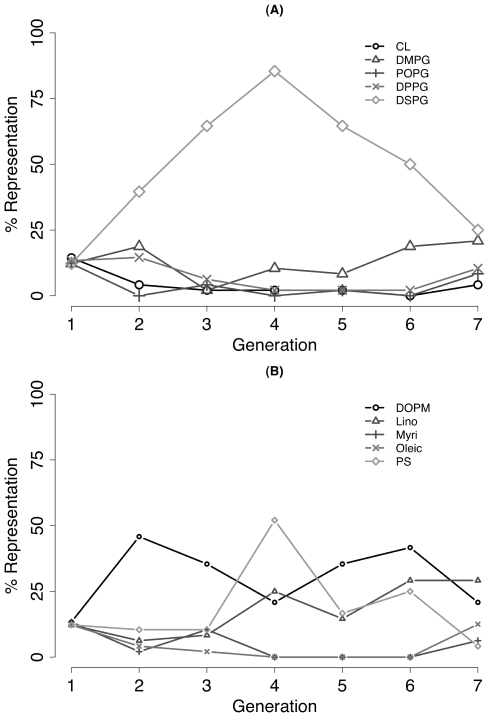
Change over time in lipid components in recipes tested. The representation of each lipid, computed as a percentage of recipes that have each particular component, for each generation of Evo-DoE is shown for seven successive generations. For generation 2–7, only model-based recipes are considered. A) The PG lipid group; B) the negatively charged lipid group. See Materials and Methods for abbreviations and groupings.

The abundance of every library component for each successive generation of Evo-DoE was recorded. The results shown in Figure 4 are for the PG lipid group and the negatively-charged lipid group (other groups not shown). In Figure 4A the strong selection for DSPG is apparent starting in the second generation while the representation of other competing lipids fluctuates. In Figure 4B, selection for DOPM, PS and linoleic acid is shown during the exploration and optimization by our protocol.

We chose a sampling of winning recipes found by Evo-DoE based on high response and diversity of components. We then analyzed this group of twelve recipes for the formation of vesicles and stability over time at varying temperatures, and normalized each result based on the standard recipe. It should be noted that we based our response function here solely upon the amount of Amphotericin B associated with the lipids and not on these secondary criteria. However, a response function could in principle combine all three criteria.

The results of the analyses of the winning recipes are shown in Table 1. Due to the selection criteria, it is not surprising that all selected recipes show better Amphotericin B incorporation (“response”) over the standard. For internal volume values that indicate the presence of liposomes, all but one of the selected recipes show lower volumes than the standard. This indicates the presence of liposomes but suggests that they are not as voluminous as liposomes prepared using the standard recipe. Surprisingly, the stability tests indicate that all of the selected recipes are more stable than the standard. We found the standard to be quite unstable and a few of our new recipes to be stable after 30 days as evidenced by the lack of aggregates in the formulations.

**Table 1:** Selected high-response formulations.

Recipe	Lipid phase	Aqueous phase	Response	Internal Volume	Stability at 4°C	Stability at 25°C	Stability at 50°C
Std	DSPG, Chol, DOPC	Succinic Acid 10 mM, pH 4.5, Na(OH), Sucrose 9%.	1.0	1.00	1.0	1.0	1.0
1	PS, Chol, SM	Bicine 100 mM, pH 8.5, Na(OH), Glucose 9%.	1.8	0.50	1.1	3.7	5.0
2	DSPG, Chol, DOPM	Bicine 100 mM, pH 8.5, Na(OH), Sucrose 4.5%.	1.7	N.A.	1.11	3.7	4.3
3	DSPG, Ergo, Lino	Mes 100 mM, pH 7, Na(OH), Sucrose 9%.	1.3	0.98	2.0	5.3	6.7
4	DSPG, Chol, DOPM	Bicine 10 mM, pH 8.5, Na(OH), Sucrose 9%.	1.8	0.52	2.0	4.7	5.0
5	DSPG, Chol, DOPM	Mes 10 mM, pH 7, Na(OH), Sucrose 4.5%.	1.7	N.A.	1.8	5.7	6.0
6	DSPG, Chol, Oleic	Bicine 100 mM, pH 8.5, Na(OH), Glucose 4.5%.	2.0	0.40	2.2	6.7	6.7
7	DMPG, Chol, DOPM	Bicine 100 mM, pH 8.5, Na(OH), Glucose 9%.	2.2	N.A.	2.0	3.0	4.7
8	DMPG, Chol, DOPM	Bicine 100 mM, pH 8.5, Na(OH), Glucose 4.5%.	1.9	0.26	1.4	3.3	5.0
9	DPPC, Ergo, SM	Bicine 10 mM, pH 8.5, Na(OH).	1.7	N.A.	2.1	6.7	5.0
10	PC, Chol, PS	Bicine 100 mM, pH 8.5, Na(OH), Glucose 9%.	2.0	0.20	2.0	5.7	5.7
11	PC, Chol, Lino	Bicine 100 mM, pH 8.5, Na(OH), Glucose 9%.	2.0	N.A.	2.1	5.7	6.7
12	DOPC, Chol, SM	Bicine 100 mM, pH 8.5, Na(OH), Glucose 4.5%.	1.9	N.A.	1.0	3.3	4.3

## Discussion

The experimental space of this system was large and complex, consisting of a 5-factor qualitative/mixture design in lipids crossed with a 7×2 factor design in the aqueous phase. There were 82,950 possible combinations, far exceeding the throughput of the experimental system. Traditional DoE methods handle such complex systems with great difficulty. They were designed to work with small numbers of experimental points when interactions among the system components are weak enough that the space can be simplified [14]. Most DoE software (e.g., Design-Expert, JMP) is not even equipped to describe this kind of system properly.

Genetic algorithms have been used in iterated high throughput optimization [1]–[5]. Their inherent stochastic nature makes them less efficient than the predictive modeling-based system presented here. Our predictive Evo-DoE algorithm is adapted for real chemical systems and was able to code the experimental system and rapidly reach an optimum.

It is important to note that the predictive model of the Evo-DoE method presented here is a statistical model built entirely from the available experimental data, and contains no expert prior knowledge regarding reactions or self-assembly processes. Neither does it contain structural information as in QSAR models [15]. Future work will consider addition of these informational components into the modeling process.

This refined method of model-based screening resulted in rapid and effective location of an optimum formulation of vesicle-encapsulated Amphotericin B. The optimum was found using only 0.5% of the total possible points, and so may be only a local optimum, but coverage of the space was nevertheless quite broad. Many formulation variations are possible and available for characterization and development into commercially viable products.

It is also possible to get a rough picture of the response landscape dictated by the lipid and aqueous phase combinations and their interactions with the target. The high dimensionality and qualitative nature of the factors makes conventional visualization difficult. We found it best to section the measured response surface to get intuitions about its topology, as shown in Figure 3.

The solutions found in our system may also be influenced by the particular high throughput method we employed, as new recipes were selected from previous ones based on how well the latter complexed with Amphotericin B using our protocol. Other protocols may place other selective factors or stresses on the system and produce different optima. However, we found many different types of recipes that can be used for Amphotericin B formulation. Other groups using different protocols have also found some of the factors that contributed to a positive response in our system. For example, the specific favorable interaction between Amphotericin B and the lipid DSPG has been known for some time, and forms the basis for the Ambisome patent (Figure 2; see [11]). However, we also found some interesting synergies among the recipe components that have not been previously described, such as the substitution of linoleic or oleic acid for the phospholipid component (typically DOPC as in the standard). Although successful formulations of Amphotericin B have been developed since the 1980s [9], there may be many different beneficial drug formulations that have yet to be discovered, some perhaps possessing better stability and pharmacokinetics.

Although our response function did not include other important parameters for drug formulation development, such as structure of the resulting particles, stability of the formulation over time, or pharmacokinetics, these could be included in a response function. The predictive Evo-DoE framework can accommodate the incorporation of such parameters. We have characterized the structure and stability of the fittest drug formulations post-optimization, and see variation in these parameters. Using our protocol with a multi-component response function would allow direct optimization of drug formulations, from the initial incorporation of drug into lipid structures through to animal models.

Our Evo-DoE methodology “closes the loop” in iterated adaptive experimentation in a high-dimensional experimental space. This closed loop can be fully automated if autonomously operating robots are used to conduct high-throughput experimentation, with the results fed directly into computers for automated statistical analysis of experimental results, and then automated intelligent design of the subsequent round of experiments could be fed directly back into the experimental robot platform. This can ultimately lead to 24/7 operation and rapid optimization of complex experimental spaces, and would mark a significant milestone in automating the scientific process [16].

The task of discovering and optimizing complex chemical systems suffers from two recurring problems: First, beneficial nonlinear interactions among system components cannot be inferred from basic chemical and physical laws, and second, as the number of system constituents and experimental parameters increases, traditional screening of experimental spaces becomes impractical and economically unfeasible. The general method applied here to optimize liposomal formulations of Amphotericin B, predictive Evo-DoE, can be used generally to discover and optimize other kinds of complex chemical systems, thus yielding a new tool for solving the problem of chemical complexity.

## Materials and Methods

### Materials

#### Aqueous components

Water, mes, phosphate buffered saline (PBS), glutamic acid (Glu), succinic acid (Succ), hepes, bicine and trizma (Tris) were purchased from Sigma Aldrich. Sodium hydroxide was purchased from Merck.

#### Solvents

Chloroform, ethanol, methanol and dimethyl sulfoxide (DMSO) were purchased from Sigma Aldrich.

#### Lipids and amphiphiles

Egg PC (L-α-phosphatidylcholine, hydrogenated), POPC (1-palmitoyl-2-oleoyl-*sn*-glycero-3-phosphocholine), DPPC (1,2-dipalmitoyl-*sn*-glycero-3-phosphocholine), DOPC (1,2-dioleoyl-*sn*-glycero-3-phosphocholine), POPG (1-palmitoyl-2-oleoyl-*sn*-glycero-3-[phospho-*rac*-(1-glycerol)]) (sodium salt), DPPG (1,2-dipalmitoyl-*sn*-glycero-3-[phospho-*rac*-(1-glycerol)]) (sodium salt), DMPG (1,2-dimyristoyl-*sn*-glycero-3-[phospho-*rac*-(1-glycerol)]) (sodium salt), DSPG (1,2-distearoyl-*sn*-glycero-3-[phospho-*rac*-(1-glycerol)]) (sodium salt) and DOPM (1,2-dioleoyl-*sn*-glycero-3-phosphomethanol) (sodium salt), were purchased from Avanti Polar Lipids. Cholesterol, ergosterol, sphingomyelin from bovine brain ≥98%, (Sphingo) 1,2-diacyl-*sn*-glycero-3-phospho-L-serine solution (PS), cardiolipin sodium salt from bovine heart (CL), oleic acid, myristoleic acid (Myri) and linoleic acid (Lino) were purchased from Sigma Aldrich.

Amphotericin B from Streptomyces ssp. in powder form and β-octylglucopyranoside (BOG) in powder form was purchased from Fluka. Sepharose 4B, Rhodamine 6G, calcein, glucose and sucrose were purchased from Sigma.

### Methods

#### Buffers preparation method

Buffers of stock solution, 100 mM, pH 4.5–7 and 8.5, NaOH were prepared and stored at 4°C. Buffer titrations were performed with a solution of 10M NaOH in water to adjust pH.

A library of 75 buffers was required to cover all possible combinations of 7 different acids at 2 concentration levels, 3 pHs, and 2 sugars at 2 concentration levels. All were stored at 4°C and used at room temperature (23°C).

#### Detergent preparation method

The BOG detergent powder was dissolved in water to a 20% (w/v) final concentration. The solution was stored at 4°C.

#### Phospholipids preparation method

Stock solutions of phospholipids in HCCl_3_, HCCl_3_/MeOH 3∶1 or HCCl_3_/MeOH 95∶5 were prepared and stored under nitrogen in Chromacol screw cap vials with silicon/teflon septa (Microcolumn Srl), and stored at −20°C. DOPC, POPC, DPPC, PC (egg), SM, PS, DOPM, oleic acid, myristoleic acid, linoleic acid, cholesterol and ergosterol were prepared at 5 mM and diluted when needed to 1 mM. CL, DPPG, POPG and DMPG were prepared at 6 mM and diluted to 0.6 mM. DSPG in powder was prepared at 0.6 mM.

#### Amphotericin B preparation method

Amphotericin B powder was dissolved in DMSO at 3.3 mM. A clear orange solution was obtained and stored at 4°C.

### Instrumentation

#### Absorbance measurements

Absorbance measurements at 415nm in the high-throughput experiment were recorded with a PerkinElmer Wallace 1420 Victor 3 Multilabel Counter, designed for 96-well plates. Absorbance spectra of the Amphotericin B in aqueous solution and in solution with vesicles were recorded with a PerkinElmer Lambda 25 spectrophotometer, using a quartz cuvette.

#### Liquid handling

The high-throughput experiment was performed with a robotic workstation for liquid handling, Xiril 75-1-2 (Switzerland). The hardware layout was designed specifically for the experimental protocol developed. The library of buffers, DMSO and BOG were contained in Chromacol 10 SV tubes and placed in a removable stainless steel tube decktray for the Xiril 75 with 96 12×75 mm positions. The dilution steps of the protocol were performed in 1.5 mL Eppendorf tubes, set in 32 position racks for 1.5 or 2 mL microfuge tubes with lids. At the end of the experiments the racks containing the buffers, DMSO and BOG were stored at 4°C. The custom racks were purchased from Xiril. The robot workstation uses Rainin tips GPS-L250 space saver 960PZ purchased from Elettrofor Sas, Rovigo, Italy.

#### High-throughput protocol

The following section describes the chemical high-throughput experiment protocol for preparing and analyzing formulations containing Amphotericin B. The DMSO solution containing Amphotericin B (crystallized at 4°C) is warmed on a standard heatblock until it becomes liquid. 2.87 µl of the Amphotericin B solution are transferred by pipette into the bottom of 500 µl volume glass vials (Chromacol 05-MTV-96) and then set in a 96-position deep well vial holder (Chromacol 05-MTP-96) from Microcolumn Srl.

The well plate containing the Amphotericin B vials is then placed in the robotic workstation and the lipids in organic solvent were added to the vials according to a well map, which specifies the exact qualitative and quantitative composition of the resulting mixture in each of the wells. At the end of the distribution step, each vial contains a mixture of three lipids and Amphotericin B.

The resulting plate containing the vials of organic solvent, lipids and Amphotericin B is then transferred into a vacuum chamber connected with a vacuum pump (KNF LAB, Pressure min 1.0 bar) with Teflon membranes. A heatblock at 100°C is set under the well plate for 20 minutes before it is removed and the evaporation process is allowed to continue for another 40 minutes, achieving a thin yellow film on the glass surface of the vials. The well plate is then transferred again to the robotic workstation and 200 µl of the hydration aqueous phase are added according to a well map. The final concentration of the lipids in the mixture is always 500 µM.

Once the lipid film is hydrated it is sonicated at 25°C for 10 minutes (Bandelin Sonorex Digitec), to promote the formation of small unilamellar vesicles (SUV). The robot then transfers 140 µl of the sonicated vesicle solution into Eppendorf tubes, prefilled with 240 µl of buffer. The samples are then centrifuged (Heraeus Biofuge Pico) at 13×10^3^ rpm for 15 minutes to separate the Amphotericin B entrapped in the formulation from free drug crystals in solution. A yellow pellet representing the free drug precipitate is visible on the bottom of the Eppendorf tubes.

178.5 µl of the supernatant is pipetted carefully into new 1.5 ml Eppendorf tubes and repositioned in the robot. To each sample, the robot adds 21 µl of DMSO and 10.5 µl of BOG, reaching a total volume of 210 µl in order to destroy any vesicles in the sample, leaving the Amphotericin B free in the organic solvent to be analyzed spectroscopically.

The robot transfers 200 µl of the resulting solution into a transparent 96-position micro-titration plate. The well plate is covered and stored at 4°C overnight. Before measuring the absorbance of the solution with the spectrophotometer, any bubbles in the samples are removed with a gentle stream of nitrogen. Triplicate absorbance measurements are made at 415 nm.

#### Stability tests

Formulations containing Amphotericin B based on twelve selected top recipes and the standard were prepared as above. The formulations were then split and incubated in parallel at 4°C, 25°C, and 50°C, over the course of 30 days. For analysis, an aliquot of 10 µl was placed on a slide with 10 µM Rhodamine 6G and analyzed with fluorescent microscopy (Nikon TE2000-S inverted fluorescence microscope with Photometrics Cascade II 512 camera and in-house software). The samples were visualized with a 40× objective and 10 different frames were captured per sample. The captured frames were then analyzed for the presence of aggregates as evidenced by small dye-containing lipid particles. They were scored as according to the criteria: score of 0 for no aggregates seen, 1 for at least one aggregate in only one captured frame, 2 for one aggregate in more than one independent frame, 3 for at least one aggregate in all frames, 4 for many aggregates in all frames. These scores were then averaged over the time course of the experiment and normalized to the standard.

#### Encapsulated volume estimation

Formulations containing Amphotericin B based on selected top recipes and the standard were prepared as above, but with 10uM calcein in the hydration buffer. The unencapsulated external dye was then separated from the lipid formulations using size exclusion chromatography (sepharose 4B column, 6×1cm). The percent dye encapsulated was then quantified on the PerkinElmer Wallace 1420 Victor 3 Multilabel Counter. The values were then normalized to the standard.

#### Response function

Response values are calculated from the absorbance of Amphotericin B after the removal of the uncomplexed Amphotericin B crystals in solution and after the destruction of the retained vesicle formulation with BOG as detergent. The Amphotericin B absorbance spectrum shows several peaks depending on the physical state of the Amphotericin B [17], [18]. Adding detergent in lipid solutions drives the micellization process and destroys the vesicles [19], which according to their large colloidal size absorb and scatter light over a broad spectrum, with a critical micellar concentration of 0.025 M [20]. By effectively cancelling out the effect of the liposomal structures and any difference in absorbance due to specific Amphotericin B-lipid interaction, absorbance peaks quantified here are determined directly from the abundance of Amphotericin B. The quantification of Amphotericin B was calculated from the absorbance of the molecule using the linear region of the titration curve.

#### Experimental design (Evo-DoE)

The full experimental space of this experiment contained 82,950 possible recipes. It consisted of all possible combinations of the following two libraries:

- An aqueous phase library (Table 2) consisting of all combinations of a buffer (15 possibilities of seven buffers at two levels, or water only) and a sugar (5 possibilities of two sugars at two levels, or no sugar), for a total of 75 possibilities.
- A lipid library (Table 3) consisting of combinations of pairs of four types of lipid (1–5 members each) with two sterols. The library was constructed using six possible combinations: (1) PC, PG, sterols (40 possibilities), (2) Negatively charged, PG, sterols (40 possibilities), (3) Sphingo, PG, sterols (10 possibilities), (4) PC, negatively charged, sterols (50 possibilities), (5) Sphingo, negatively charged, sterols (10 possibilities), or (6) PC, Sphingo, sterols (8 possibilities), for a total of 158 possibilities.

**Table 2:** Aqueous phase library.

Buffers	pH group	Levels (mM)	Sugars	Levels (%)
HEPES	7	10, 100	Glucose	4.5, 9
MES	7	10, 100	Sucrose	4.5, 9
Glutamic acid	8.5	10, 100	No Sugar	
Succinic acid	4.5	10, 100		
Bicine	8.5	10, 100		
Tris	8.5	10, 100		
PBS	7	10, 100		
No buffer				

**Table 3:** Lipid library.

Lipid Categories	Lipids
PC	DOPC, POPC, DPPC, PC (egg)
PG	CL, DSPG, DPPG, POPG, DMPG
Sterol	Cholesterol, Ergosterol
Negatively charged	PS, DOPM, oleic acid, myristoleic acid, linoleic acid
Sphingo	Sphingomyelin

Because the lipid combinations form a mixture system, the range of quantitative possibilities (relative concentrations of each of the lipid types) is a polytope. The polytope and its extreme vertices were generated using JMP software, and a set of the four vertices, two midpoints, and the overall centroid were selected for the experiments, for a total of seven concentration profiles for each of the 158 lipid combinations.

Thus, the total number of possible combinations is given by 75 (buffers) * 158 (qualitative lipid mixtures) * 7 (quantitative lipid possibilities) = 82,950 (total experimental space).

Applying conventional DoE methods to a complex system like this is very difficult. We attempted to generate a screening design using two major software packages (JMP and Design-Expert) and found them incapable of dealing with multilevel qualitative factors with the sorts of constraints specified for the lipid library.

Experimentation using iterated high-throughput screening and Evo-DoE begins with an initial generation selected from the whole experimental space. In standard methods such as genetic algorithms, this selection is random. More recently, low-discrepancy random sequence protocols have been used to avoid the gaps and overlapping points that frequently arise in the random approach, by biasing the random sampling toward unsampled regions of the experimental space. This is essentially a high-dimensional version of the stochastic sampling used in Kriging [7]. We further refined the initial generation using the concept of incomplete factorial experiments, by using a balanced design as the first generation [21], [22]. Our results from the earlier experiments and simulations have shown that two-way interactions appear to form the “ridges” our algorithm explores to find the peaks; this balanced design samples those ridges effectively. The recipe set was generated by successively eliminating duplicative runs from a moderately large number of initial random recipes. In subsequent generations 80–90% of the points were selected by predictive modeling, and the rest by a stochastic sampling algorithm described above.

Predictions of experiments were obtained from a neural network model (learned with back-propagation using *nnet* in the R language after standardizing all inputs and normalizing the output to the [0,1] interval) with 28 inputs and one output (levels “No buffer” and “No sugar” in the aqueous phase were regarded as two separate input variables, each taking on either zero or one). Each neural network was constructed with particular metaparameter values (weight decay and number of hidden nodes). The model's metaparameters were selected using a bagging process, repeating the model learning on a number of different data sets, each being a different random sample of the observed experiments, and a number of times on each data set. Each configuration of metaparameters was then assigned a quality measure, calculated as the median correlation between observations and predictions over all the repeats.
